# Supplementary figures and images for: Biodiversity of marine microbes is safeguarded by phenotypic heterogeneity in ecological traits
Source: PLoS One. 2021 Aug 4;16(8):e0254799. doi: 10.1371/journal.pone.0254799 (PMC8336841; doi:10.1371/journal.pone.0254799)

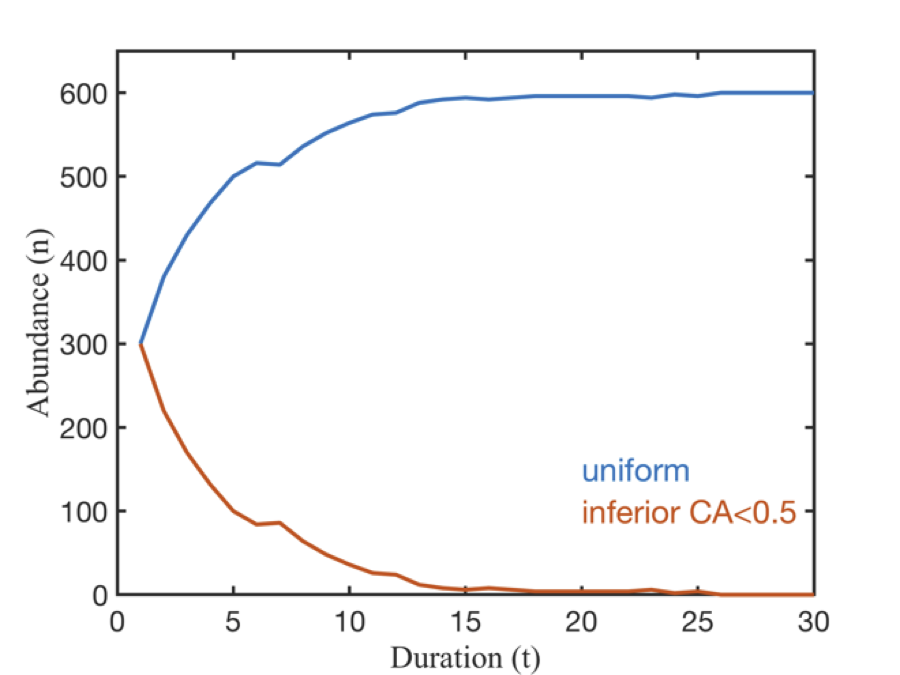

Supplement: S1 Fig — Competition between uniform (blue) and an inferior competitor (orange) that has mean competitive ability < 0.5. In this case, our model faithfully reproduces the ‘competitive exclusion’ principle [39], and the species with the lower mean is eliminated. (TIF) [file pone.0254799.s001.tif]

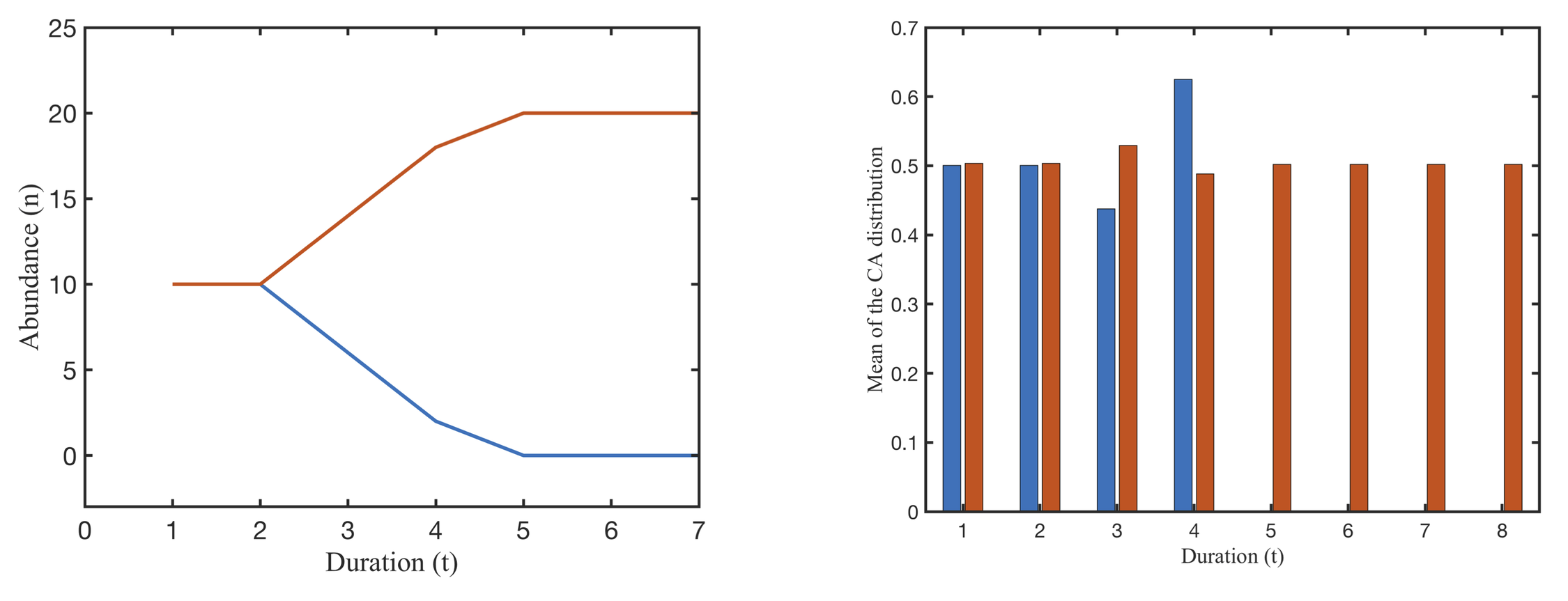

Supplement: S2 Fig — On the left, the nominally uniform distribution goes extinct when the uniform distribution is statistically poorly characterized because few individuals represent the distribution. The right shows the mean competitive ability of the two cohorts in this simulation. The inferior mean in time step 3 results in extinction of the blue type. Even a reversal of fortunes in the next time step, with a higher mean CA for blue than orange does not balance the competition, and the blue population is eliminated. The blue-uniform strategy could have persisted longer by chance alone, but the randomly selected orange competitors all had superior competitive ability due to low population abundances. The trivial example of competition with an inferior competitor, characterized by a lower mean competitive ability, leads to extinction as shown in S1 Fig. At low population sizes the underlying strategy distributions can be poorly represented, especially for the maximally variable uniform distribution as shown in S2 Fig. In these cases, extinctions can be observed. Such dynamics would apply when few individuals colonize a new habitat, mutations yield new functions or other events when a few individuals represent the entirety of the population. (TIF) [file pone.0254799.s002.tif]
