# Supplementary material for: Biodiversity of marine microbes is safeguarded by phenotypic heterogeneity in ecological traits
Source: PLoS One. 2021 Aug 4;16(8):e0254799. doi: 10.1371/journal.pone.0254799 (PMC8336841; doi:10.1371/journal.pone.0254799)
Supplement: S1 Appendix — (ZIP) [file pone.0254799.s003.zip › S1_Appendix.pdf]

S1 Mathematical proofs for the discrete model. We determine the equilibrium strategies by proving several propositions, building up from the case of two competing strategies to arbitrarily many competing strategies. The techniques we use to prove Theorem 1 are quite different in the discrete and continuous cases. In both cases, however, we use the same method of building from the special case of two competing species and use this case to establish our theorem for arbitrary and possibly fluctuating numbers of competing species. This technique bears some resemblance to a proof by induction, viewing the 'base case' as the case of two competing species.

Proposition 1. *A* uniform *strategy is defined for $|U| > 0$ to satisfy $U(x_j) = \frac{|U|}{M+1}$ for all j. Then for any strategy A, with $|A| > 0$ and $\mathrm{MCA}(A) \leq \frac{1}{2}$, we have*

$$\wp(A;U) \leq 0, \quad \wp(U;A) \geq 0,$$

with equality if and only if $\mathrm{MCA}(A) = \frac{1}{2}$.

Proof. The payoff

$$\wp(A;U) = \frac{1}{|A|+|U|} \sum_{j=0}^{M} A(x_j) \left(\sum_{i<j} U(x_i) - \sum_{i>j} U(x_i) \right).$$

We note that

$$\sum_{i>j} U(x_i) = |U| - U(x_j) - \sum_{i<j} U(x_i) = |U| - \frac{|U|}{M+1} - j\frac{|U|}{M+1},$$

so the payoff is

$$\begin{aligned}
\wp(A;U) &= \frac{1}{|A|+|U|} \sum_{j=0}^{M} A(x_j) \left(2j\frac{|U|}{M+1} - |U| + \frac{|U|}{M+1} \right) \\
&= \frac{1}{|A|+|U|} \left(\frac{2M|U|}{M+1} \sum_{j=0}^{M} A(x_j) \frac{j}{M} - |U||A| + \frac{|U||A|}{M+1} \right) \\
&= \frac{1}{|A|+|U|} \left(\frac{2M|A||U|}{M+1} \mathrm{MCA}(A) - |U||A| + \frac{|U||A|}{M+1} \right) \\
&= \frac{|A||U|}{(M+1)(|A|+|U|)} \left(2M\,\mathrm{MCA}(A) - (M+1) + 1 \right) \\
&= \frac{M|A||U|}{(M+1)(|A|+|U|)} \left(2\,\mathrm{MCA}(A) - 1 \right) \leq 0 \text{ since } \mathrm{MCA}(A) \leq \frac{1}{2},
\end{aligned}$$

and we see that equality holds precisely when $\mathrm{MCA}(A) = \frac{1}{2}$. □

Proposition 2. *Assume that a strategy A in the discrete case has* $\mathrm{MCA}(A) = 0.5$ *and is not symmetric about* 0.5*, then there exists at least one ℓ with $0 \leq \ell \leq M$ such that*

$$A(x_\ell) + 2\sum_{j<\ell} A(x_j) > A(x_{M-\ell}) + 2\sum_{j>M-\ell} A(x_j).$$

Proof. The proof is by contrapositive. We shall assume that no such ℓ exists. Then, we will show that the strategy, A, is symmetric about the competitive ability 0.5. So, we assume that for all ℓ we have the inequality:

$$A(x_\ell) + 2\sum_{j<\ell} A(x_j) \leq A(x_{M-\ell}) + 2\sum_{j>M-\ell} A(x_j). \tag{5}$$

Whenever the sum is empty, we define its value to be equal to zero. We claim that this inequality must be an equality for all ℓ. To see this, we shall sum over all ℓ. On the left we obtain

$$\sum_{\ell=0}^{M} 2\sum_{j<\ell} A(x_j) + \sum_{\ell=0}^{M} A(x_\ell) = \sum_{k=0}^{M} [2(M-k)+1]A(x_k).$$

On the right,

$$\sum_{\ell=0}^{M} 2\sum_{j>M-\ell} A(x_j) + \sum_{\ell=0}^{M} A(x_{M-\ell}) = \sum_{k=0}^{M} (2k+1)A(x_k).$$

Hence, we have the inequality

$$\sum_{k=0}^{M} [2(M-k)+1]A(x_k) \leq \sum_{k=0}^{M} (2k+1)A(x_k).$$

This reduces to

$$\sum_{k=0}^{M} 2MA(x_k) = 2M|A| \leq \sum_{k=0}^{M} 4kA(x_k).$$

Since the MCA of A is assumed to be equal to $\frac{1}{2}$,

$$\sum_{k=0}^{M} 4kA(x_k) = 4M\frac{|A|}{2} = 2M|A|.$$

Hence, both sides are equal. Therefore, the inequality (5) can never be strict, because once we sum over all $0 \leq \ell \leq M$, the result is an equality. Hence, we have for all $0 \leq \ell \leq M$,

$$2\sum_{j<\ell} A(x_j) + A(x_\ell) = 2\sum_{j>M-\ell} A(x_j) + A(x_{M-\ell}). \tag{6}$$

We shall prove by induction on ℓ that A is symmetric about 0.5, which will complete the proof by contrapositive. For the base case, $\ell = 0$, so the left side of (6) is $A(x_0)$, and the right side is $A(x_M)$, and these must be equal. Next, we inductively assume that $A(x_j) = A(x_{M-j})$ holds true for all $j = 0, \ldots, \ell$ for some $\ell \geq 0$ with $\ell \leq M-1$. Then, for $\ell + 1 \leq M$ we have by (6)

$$2\sum_{j<\ell+1} A(x_j) + A(x_{\ell+1}) = 2\sum_{j>M-\ell-1} A(x_j) + A(x_{M-\ell-1}).$$

Since

$$2\sum_{j<\ell} A(x_j) + A(x_\ell) = 2\sum_{j>M-\ell} A(x_j) + A(x_{M-\ell}),$$

$$2A(x_\ell) + A(x_{\ell+1}) = 2A(x_{M-\ell}) + A(x_{M-\ell-1}).$$

By the induction assumption, $A(x_\ell) = A(x_{M-\ell})$, so we obtain $A(x_{\ell+1}) = A(x_{M-\ell-1})$. Consequently, by induction, we have proven that A This completes the proof by induction. Consequently, we obtain that A is symmetric about 0.5, which is a contradiction to the hypothesis of the proposition. This completes the proof by contrapositive. □

We use the preceding proposition to prove that strategies that are not symmetric about $\frac{1}{2}$ can be defeated. First we note that for all strategies, there is a zero-sum dynamic, namely,

$$\wp(A; B) + \wp(B; A) = 0.$$

Proposition 3. *Let A be a strategy that has* $\mathrm{MCA}(A) = \frac{1}{2}$ *that is not symmetric with respect to $\frac{1}{2}$. Then there exists a strategy B that has* $\mathrm{MCA}(B) = \frac{1}{2}$ *for which*

$$\wp(A; B) < 0, \quad \wp(B; A) > 0.$$

Proof. By the preceding proposition, since A is not symmetric with respect to 0.5, we have proven that there is an ℓ such that

$$2\sum_{j<\ell} A(x_j) + A(x_\ell) > 2\sum_{j>M-\ell} A(x_j) + A(x_{M-\ell}). \tag{7}$$

Whenever the sum is empty, define its value to be zero. We define

$$B(x_j) := \begin{cases} \frac{|A|}{2} & j \in \{\ell, M-\ell\} \\ 0 & \text{otherwise.}\} \end{cases}$$

The mean competitive ability of B,

$$\mathrm{MCA}(B) = \frac{1}{|A|}\left(\frac{\ell}{M}\frac{|A|}{2} + \frac{M-\ell}{M}\frac{|A|}{2}\right) = \frac{1}{2}.$$

We compute the payoff

$$\wp(B; A) =$$

$$\frac{1}{2|A|}\left(B(x_\ell)\left(\sum_{j<\ell} A(x_j) - \sum_{j>\ell} A(x_j)\right) + B(x_{M-\ell})\left(\sum_{j<M-\ell} A(x_j) - \sum_{j>M-\ell} A(x_j)\right)\right)$$

$$= \frac{1}{4}\left(\left(\sum_{j<\ell} A(x_j) - \sum_{j>\ell} A(x_j)\right) + \left(\sum_{j<M-\ell} A(x_j) - \sum_{j>M-\ell} A(x_j)\right)\right).$$

We note that

$$\sum_{j>\ell} A(x_j) = |A| - A(x_\ell) - \sum_{j<\ell} A(x_j), \quad \sum_{j<M-\ell} A(x_j) = |A| - A(x_{M-\ell}) - \sum_{j>M-\ell} A(x_j).$$

This allows us to re-write $\wp(B;A)$

$$= \frac{1}{4}\left(\sum_{j<\ell} A(x_j) - \left(|A| - A(x_\ell) - \sum_{j<\ell} A(x_j)\right)\right)$$

$$+ \frac{1}{4}\left(|A| - A(x_{M-\ell}) - \sum_{j>M-\ell} A(x_j) - \sum_{j>M-\ell} A(x_j)\right)$$

$$= \frac{1}{4}\left(2\sum_{j<\ell} A(x_j) + A(x_\ell) - \left(2\sum_{j>M-\ell} A(x_j) + A(x_{M-\ell})\right)\right) > 0,$$

with the final inequality following from (7). □

Proposition 4. *Assume that (A, B) is an equilibrium point. Then*

$$\wp(A;B) = \wp(B;A) = 0.$$

Moreover, we have for any strategy C,

$$\wp(*;C) \geq 0, \quad * = A, B. \tag{8}$$

In case M is odd, all equilibrium strategies are uniform. In case M is even, all equilibrium strategies have MCA *equal to $\frac{1}{2}$ and further satisfy $A(x_{2j}) = A(x_0)$, $A(x_{2j+1}) = A(x_1)$ for all $j = 0, 1, \ldots, \frac{M}{2}$. Furthermore equality holds in* (8) *if and only if* $\mathrm{MCA}(C) = \frac{1}{2}$.

Proof. To prove the first statement, we note that if

$$\wp(A;B) < 0 \implies \wp(B;B) = 0 > \wp(A;B),$$

contradicting the definition of equilibrium strategy. Thus $\wp(A;B) \geq 0$. The same argument shows that $\wp(A;B) \geq 0$, so by the zero-sum dynamic, $\wp(A;B) = \wp(B;A) = 0$. To prove the second statement, assume that there is a strategy C such that

$$\wp(A;C) < 0.$$

Then, by the zero-sum dynamic

$$\wp(C;A) > 0 = \wp(B;A),$$

contradicting the definition of B as an equilibrium strategy. The same argument proves that (8) holds for B as well. By Proposition 1, any strategy with MCA $< \frac{1}{2}$ is not an equilibrium strategy. By Proposition 3, any strategy that is not symmetric with respect to $\frac{1}{2}$ is not an equilibrium strategy. By Proposition 1, uniform strategies U_1 and U_2 satisfy

$$\wp(U_1;U_2) = 0 = \wp(U_2;U_1), \quad \wp(C;U_i) = -\wp(U_i;C) \leq 0,$$

for any strategy C, with equality if and only if $\mathrm{MCA}(C) = \frac{1}{2}$. Consequently, any such (U_1, U_2) is an equilibrium point. Let us now see that in case M is odd, all equilibrium strategies are uniform strategies. For this aim, it suffices to consider strategies that have MCA $= \frac{1}{2}$ and are symmetric about $\frac{1}{2}$. We will show that such a strategy A that is not uniform cannot be an equilibrium strategy. Let ℓ be the smallest integer such that $A(x_{\ell+1}) = \ldots = A(x_{M-\ell-1})$. Since we have assumed that A is symmetric,

$$A\left(x_{\frac{M-1}{2}}\right) = A\left(x_{\frac{M+1}{2}}\right).$$

Consequently $\ell < \frac{M-1}{2}$, and by the assumption that A is not uniform, there exists such an $\ell \geq 0$. There are only two cases to consider:

$$\text{Case 1: } A(x_\ell) > A(x_{\ell+1}), \quad \text{Case 2: } A(x_\ell) < A(x_{\ell+1}).$$

Assume first that we are in the first case. Let

$$B(x_j) := \begin{cases} |A|\frac{M-2\ell}{M-2\ell+1} & j = \frac{M-1}{2}, \\ |A|\frac{1}{M-2\ell+1} & j = M-\ell, \\ 0 & \text{otherwise.} \end{cases}$$

Then we compute

$$\begin{aligned} \text{MCA}(B) &= \frac{M-1}{2M}\frac{M-2\ell}{M-2\ell+1} + \frac{M-\ell}{M}\frac{1}{M-2\ell+1} \\ &= \frac{(M-1)(M-2\ell)+2(M-\ell)}{2M(M-2\ell+1)} = \frac{1}{2}. \end{aligned}$$

We compute the payoff $\wp(B; A) =$

$$\frac{1}{2|A|}|A|\frac{M-2\ell}{M-2\ell+1}\left(\sum_{i<\frac{M-1}{2}} A(x_i) - \sum_{i>\frac{M-1}{2}} A(x_i)\right)$$

$$+\frac{1}{2|A|}|A|\frac{1}{M-2\ell+1}\left(\sum_{i<M-\ell} A(x_i) - \sum_{i>M-\ell} A(x_i)\right).$$

$$= \frac{1}{2(M-2\ell+1)}\left((M-2\ell)\left(\sum_{i<\frac{M-1}{2}} A(x_i) - \sum_{i>\frac{M-1}{2}} A(x_i)\right)\right)$$

$$+\frac{1}{2(M-2\ell+1)}\left(\sum_{i<M-\ell} A(x_i) - \sum_{i>M-\ell} A(x_i)\right).$$

By the symmetry assumption, this is

$$= \frac{1}{2(M-2\ell+1)}\left(-(M-2\ell)A(x_{\frac{M+1}{2}}) + \left(\sum_{i<M-\ell} A(x_i) - \sum_{i>M-\ell} A(x_i)\right)\right).$$

By the definition of ℓ, this is

$$= \frac{1}{2(M-2\ell+1)}\left(-(M-2\ell)A(x_{\ell+1}) + \left(\sum_{i<M-\ell} A(x_i) - \sum_{i>M-\ell} A(x_i)\right)\right).$$

Using the symmetry of A and the definition of ℓ this is

$$= \frac{1}{2(M-2\ell+1)}\left(-(M-2\ell)A(x_{\ell+1}) + (M-2\ell-1)A(x_{\ell+1}) + A(x_\ell)\right)$$

$$= \frac{1}{2(M-2\ell+1)}(A(x_\ell) - A(x_{\ell+1})) > 0,$$

with the last inequality following from the fact that we are in Case 1.

Next we assume that we are in Case 2, so we have $A(x_\ell) < A(x_{\ell+1})$. We define

$$B(x_j) := \begin{cases} |A|\frac{1}{M-2\ell+1} & j=\ell, \\ |A|\frac{M-2\ell}{M-2\ell+1} & j=\frac{M+1}{2}, \\ 0 & \text{otherwise.} \end{cases}$$

Then we compute

$$\mathrm{MCA}(B) = \frac{\ell}{M}\frac{1}{M-2\ell+1} + \frac{M+1}{2M}\frac{M-2\ell}{M-2\ell+1} = \frac{2\ell+(M+1)(M-2\ell)}{2M(M-2\ell+1)} = \frac{1}{2}.$$

By definition, $\wp(B;A) =$

$$\frac{1}{2|A|}|A|\frac{1}{M-2\ell+1}\left(\sum_{j<\ell}A(x_j) - \sum_{j>\ell}A(x_j)\right)$$

$$+\frac{1}{2|A|}|A|\frac{M-2\ell}{M-2\ell+1}\left(\sum_{j<\frac{M+1}{2}}A(x_j) - \sum_{j>\frac{M+1}{2}}A(x_j)\right).$$

$$= \frac{1}{2(M-2\ell+1)}\left(-(M-2\ell-1)A(x_{\ell+1}) - A(x_\ell) + (M-2\ell)A(x_{\frac{M-1}{2}})\right)$$

$$= \frac{1}{2(M-2\ell+1)}\left(-(M-2\ell-1)A(x_{\ell+1}) - A(x_\ell) + (M-2\ell)A(x_{\ell+1})\right)$$

$$= \frac{1}{2(M-2\ell+1)}(A(x_{\ell+1}) - A(x_\ell)) > 0.$$

Above we have used the symmetry of A and the definition of ℓ, and the fact that we are in Case 2. This completes the proof that in case M is odd, all equilibrium strategies are uniform strategies, and all equilibrium points consist of uniform strategies.

Let us now assume that M is even. In this case we already have demonstrated that equilibrium strategies must have $\mathrm{MCA} = \frac{1}{2}$ and must be symmetric. The last part of the proof is to demonstrate that in this case, equilibrium strategies must further satisfy the condition $A(x_{2j}) = A(x_0)$ and $A(x_{2j+1}) = A(x_1)$ for all $1 \le j \le \frac{M}{2}$. This will proceed in two parts. First we show that a strategy that has $\mathrm{MCA} = \frac{1}{2}$, is symmetric, but does not satisfy this condition cannot be an equilibrium strategy. We therefore assume that there is some $2 \le \ell \le \frac{M}{2}$ such that $A(x_{\ell-2}) \neq A(x_\ell)$. There are two cases:

$$\text{Case 1: } A(x_{\ell-2}) < A(x_\ell), \qquad \text{Case 2: } A(x_{\ell-2}) > A(x_\ell).$$

Assume we are in the first case. We define

$$B(x_j) := \begin{cases} \frac{|A|}{2} & j = M-\ell+1, \\ \frac{|A|}{4} & j=\ell, \\ \frac{|A|}{4} & j=\ell-2. \end{cases}$$

Then

$$\mathrm{MCA}(B) = \frac{1}{|A|}\left(\frac{|A|}{2}\frac{M-\ell+1}{M} + \frac{|A|}{4}\frac{\ell}{M} + \frac{|A|}{4}\frac{\ell-2}{M}\right)$$

$$= \frac{2M-2\ell+2+\ell+\ell-2}{4M} = \frac{1}{2}.$$

The payoff $\wp(B;A) =$

$$\frac{1}{2|A|}\frac{|A|}{4}\left(\sum_{j<\ell-2}A(x_j)-\sum_{j>\ell-2}A(x_j)+\sum_{j<\ell}A(x_j)-\sum_{j>\ell}A(x_j)\right)$$
$$+\frac{1}{2|A|}\frac{|A|}{2}\left(\sum_{j<M-\ell+1}A(x_j)-\sum_{j>M-\ell+1}A(x_j)\right)$$

$$=\frac{1}{8}\left(\sum_{j<\ell-2}A(x_j)-A(x_{\ell-1})-A(x_\ell)-\sum_{\ell<j\leq M-\ell}A(x_j)-A(x_{M-\ell+1})-A(x_{M-\ell+2})\right)$$
$$+\frac{1}{8}\left(-\sum_{j>M-\ell+2}A(x_j)+\sum_{j<\ell}A(x_j)-\sum_{\ell<j\leq M-\ell}A(x_j)-\sum_{j>M-\ell}A(x_j)\right)$$
$$+\frac{1}{4}\left(\sum_{j<\ell-1}A(x_j)+A(x_{\ell-1})+A(x_\ell)+\sum_{\ell<j\leq M-\ell}A(x_j)-\sum_{j>M-\ell+1}A(x_j)\right).$$

Note that by symmetry, $A(x_j) = A(x_{M-j})$. Therefore this is

$$\frac{1}{8}\left(-2A(x_{\ell-1})-A(x_\ell)-A(x_{\ell-2})-\sum_{\ell<j\leq M-\ell}A(x_j)-\sum_{\ell<j\leq M-\ell}A(x_j)\right)$$
$$+\frac{1}{4}\left(A(x_{\ell-1})+A(x_\ell)+\sum_{\ell<j\leq M-\ell}A(x_j)\right)=\frac{1}{8}\left(A(x_\ell)-A(x_{\ell-2})\right)>0.$$

The last inequality follows because we are in Case 1. Now let us assume that we are in Case 2, so $A(x_{\ell-2}) > A(x_\ell)$. In this case we define

$$B(x_j)=\begin{cases}\frac{|A|}{2} & j=\ell-1,\\ \frac{|A|}{4} & j=M-\ell,\\ \frac{|A|}{4} & j=M-\ell+2.\end{cases}$$

Then,

$$\mathrm{MCA}(B)=\frac{1}{|A|}\left(\frac{|A|}{2}\frac{\ell-1}{M}+\frac{|A|}{4}\frac{M-\ell}{M}+\frac{|A|}{4}\frac{M-\ell+2}{M}\right)$$
$$=\frac{2\ell-2+M-\ell+M-\ell+2}{4M}=\frac{1}{2}.$$

We compute that the payoff $\wp(B;A) =$

$$\frac{1}{2|A|}\frac{|A|}{2}\left(\sum_{j<\ell-1}A(x_j)-\sum_{\ell\leq j<M-\ell}A(x_j)-A(x_{M-\ell})-A(x_{M-\ell+1})-\sum_{j>M-\ell+1}A(x_j)\right)$$

$$+\frac{1}{2|A|}\frac{|A|}{4}\left(\sum_{j<\ell}A(x_j)+\sum_{\ell\le j<M-\ell}A(x_j)-\sum_{j>M-\ell}A(x_j)\right)$$

$$+\frac{1}{2|A|}\frac{|A|}{4}\left(\sum_{j<\ell-2}A(x_j)+A(x_{\ell-2})+A(x_{\ell-1})\right)$$

$$+\frac{1}{2|A|}\frac{|A|}{4}\left(\sum_{\ell\le j<M-\ell}A(x_j)+A(x_{M-\ell})+A(x_{M-\ell+1})\right)$$

$$-\frac{1}{2|A|}\frac{|A|}{4}\sum_{j>M-\ell+2}A(x_j).$$

By the symmetry, $A(x_j)=A(x_{M-j})$ and so this is

$$\frac{1}{4}\left(-\sum_{\ell\le j<M-\ell}A(x_j)-A(x_\ell)-A(x_{\ell-1})\right)$$

$$+\frac{1}{8}\left(\sum_{\ell\le j<M-\ell}A(x_j)+A(x_{\ell-2})+A(x_\ell)+2A(x_{\ell-1})+\sum_{\ell\le j<M-\ell}A(x_j)\right)$$

$$=\frac{1}{8}\left(A(x_{\ell-2})-A(x_\ell)\right)>0.$$

The last inequality follows because we are in Case 2.

To complete the proof, we will demonstrate that $\wp(A;B)\ge 0$ for all strategies B for any strategy A that satisfies:

1. $\mathrm{MCA}(A)=\frac{1}{2}$;
2. $A(x_j)=A(x_{M-j})$ for all j;
3. for all $2\le j\le \frac{M}{2}$, $A(x_j)=A(x_{j-2})$.

Moreover, we will prove that $\wp(A;B)=0$ if and only if $\mathrm{MCA}(B)=\frac{1}{2}$. Consequently, in case M is even, all equilibrium strategies are of this type. Assuming that A satisfies the conditions above, there exist non-negative numbers, a and b such that

$$A(x_{2j})=a,\quad A(x_{2j+1})=b,\quad \text{for all } j=0,\dots,\frac{M}{2}.$$

We shall compute the payoff for an arbitrary strategy B in competition against such a strategy A,

$$\wp(B;A)=\frac{1}{|A|+|B|}\sum_{i=0}^{M}B(x_i)\left(\sum_{j=0}^{i-1}A(x_j)-\sum_{j=i+1}^{M}A(x_j)\right).$$

To exploit the symmetry of A, we write this as

$$\frac{1}{|A|+|B|}\sum_{i=0}^{M/2}B(x_i)\left(\sum_{j=0}^{i-1}A(x_j)-\sum_{j=i+1}^{M}A(x_j)\right)$$

$$+\frac{1}{|A|+|B|}\sum_{i=\frac{M}{2}+1}^{M} B(x_i)\left(\sum_{j=0}^{i-1} A(x_j) - \sum_{j=i+1}^{M} A(x_j)\right)$$

$$= -\frac{1}{|A|+|B|}\sum_{i=0}^{M/2} B(x_i)\sum_{j=i+1}^{M-i} A(x_j) + \frac{1}{|A|+|B|}\sum_{i=\frac{M}{2}+1}^{M} B(x_i)\sum_{j=M-i}^{i-1} A(x_j),$$

having used the symmetry of A above. The first sum over j, $\sum_{j=i+1}^{M-i} A(x_j)$, has $M-2i$ summands, half of which are equal to a, and half of which are equal to b. Similarly, the second sum over j, $\sum_{j=M-i}^{i-1} A(x_j)$, has $2i-M$ summands, half of which are equal to a, and half of which are equal to b. Therefore we obtain:

$$\wp(B;A) = \frac{1}{|A|+|B|}\left[-\sum_{i=0}^{M/2} B(x_i)\left(\frac{M}{2}-i\right)(a+b) + \sum_{i=\frac{M}{2}+1}^{M} B(x_i)\left(i-\frac{M}{2}\right)(a+b)\right]$$

$$= \frac{1}{|A|+|B|}(a+b)\sum_{i=0}^{M} B(x_i)\left(i-\frac{M}{2}\right) = \frac{M(a+b)}{|A|+|B|}\left(-\frac{|B|}{2}+\sum_{i=0}^{M}\frac{i}{M}B(x_i)\right)$$

$$= \frac{M(a+b)}{|A|+|B|}\left(-\frac{|B|}{2}+|B|\,\mathrm{MCA}(B)\right) \le 0,$$

with equality if and only if $\mathrm{MCA}(B)=\frac{1}{2}$. Consequently we have proven that for such an A, we have

$$\wp(B;A) \le 0 \text{ for any } B \text{ with equality if and only if } \mathrm{MCA}(B)=\frac{1}{2}$$

that immediately implies

$$\wp(A;B) \ge 0 \text{ for any } B \text{ with equality if and only if } \mathrm{MCA}(B)=\frac{1}{2}.$$

We therefore obtain that in case M is even, equilibrium strategies are precisely those that satisfy these three conditions. □

We will now use the results obtained here to determine all equilibrium strategies in the discrete model for arbitrary numbers of competing species.

Proof of Theorem 1 for the discrete model

Proof. We will first show that all strategies that are equilibrium strategies for two competing species are also equilibrium strategies for any number of competing species. So, assume that $\{A_k\}_{k=1}^n$ are a set of strategies that satisfy

$$\wp(A_k;B) \ge 0$$

for all strategies B as in Definition 1, for all $k=1,\ldots,n$. Then, we have

$$\wp(A_k;A_j)\ge 0, \quad \wp(A_j;A_k)\ge 0 \implies \wp(A_k;A_j)=0, \quad \forall k,j\in\{1,2,\ldots,n\}.$$

Above we have used the zero-sum dynamic. Consequently, we have

$$\sum_{j=0}^{M} A_k(x_j) \left[\sum_{i<j} A_\ell(x_i) - \sum_{i>j} A_\ell(x_i) \right] = 0, \quad \forall k, \ell \in \{1, \ldots, n\},$$

and therefore

$$\wp(A_k; A_1, \ldots, A_{k-1}, A_{k+1}, A_n) = 0 \quad \forall k = 1, \ldots, n.$$

Now, consider any strategy B as in Definition 1. Then if we change strategy A_1 to B, the resulting payoff $\wp(B; A_2, \ldots, A_n) =$

$$\frac{1}{|B| + \sum_{k\geq 2} |A_k|} \sum_{j=0}^{M} B(x_j) \left[\sum_{i<j} \left(B(x_i) + \sum_{k\geq 2} A_k(x_i) \right) - \sum_{i>j} \left(B(x_i) + \sum_{k\geq 2} A_k(x_i) \right) \right].$$

However, by the assumption on A_k and the zero-sum dynamic, we have

$$\wp(A_k; B) \geq 0 \implies \wp(B; A_k) \leq 0 \implies \sum_{j=0}^{M} B(x_j) \left[\sum_{i<j} A_k(x_i) - \sum_{i>j} A_k(x_i) \right] \leq 0,$$

for all $k = 2, \ldots, n$. Therefore, since the internal competition within B does not affect the payoff, we have

$$\sum_{j=0}^{M} B(x_j) \left[\sum_{i<j} \sum_{k=2}^{n} A_k(x_i) - \sum_{i>j} \sum_{k=2}^{n} A_k(x_i) \right] \leq 0 \implies \wp(B; A_2, \ldots, A_n) \leq 0. \quad (9)$$

By (9), we therefore have $\wp(B; A_2, \ldots, A_n) \leq \wp(A_1; A_2, \ldots, A_n) = 0$. The same argument applies to A_k for all k, namely, for any B as in, Definition 1,

$$\wp(B; A_1, \ldots, A_{k-1}, A_{k+1}, \ldots A_n) \leq 0 = \wp(A_k; A_1, \ldots, A_{k-1}, A_{k+1}, \ldots, A_n).$$

It follows that the set of strategies $\{A_k\}_{k=1}^n$ is an equilibrium point.

To prove the converse, we begin by assuming that the set of strategies $\{A_k\}_{k=1}^n$ comprise an equilibrium point. For the sake of contradiction, let us see what would happen if

$$\wp(A_1; A_2 \ldots, A_n) < 0 \implies \sum_{j=0}^{M} A_1(x_j) \left[\sum_{i<j} \sum_{k=2}^{n} A_k(x_i) - \sum_{i<j} \sum_{k=2}^{n} A_k(x_i) \right] < 0.$$

We define a new strategy B_1,

$$B_1(x_j) := \sum_{k=2}^{n} A_k(x_j) \implies \mathrm{MCA}(B_1) \leq \frac{1}{2},$$

and

$$\sum_{j=0}^{M} B_1(x_j) \left[\sum_{i<j} \sum_{k=2}^{n} A_k(x_i) - \sum_{i>j} \sum_{k=2}^{n} A_k(x_i) \right] = 0$$

$$\implies \wp(B_1; A_2, \ldots, A_n) = 0 > \wp(A_1; \ldots, A_n),$$

contradicting the definition of equilibrium strategy. We therefore have that $\wp(A_1; A_2, \ldots, A_n) \geq 0$, and the same argument shows that $\wp(A_k; A_1, \ldots, A_{k-1}, A_{k+1}, \ldots A_n) \geq 0$ for all $k = 1, \ldots, n$. By the zero-sum dynamic, we also have

$$\sum_{k=1}^{n} \wp(A_k; \ldots) = 0 \implies \wp(A_k; \ldots) = 0, \quad \forall k = 1, 2, \ldots, n.$$

By the definition of equilibrium strategy, we must have that for any strategy B,

$$\wp(B; A_2, \ldots, A_n) \leq \wp(A_1; A_2 \ldots, A_n) = 0$$
$$\implies \sum_{j=0}^{M} B(x_j) \left[\sum_{i<j} \sum_{k=2}^{n} A_k(x_i) - \sum_{i>j} \sum_{k=2}^{n} A_k(x_i) \right] \leq 0.$$

Then the payoff to any strategy B in competition with B_1 satisfies

$$\wp(B; B_1) \leq 0.$$

By the zero sum dynamic, we therefore have

$$\wp(B_1; B) \geq 0.$$

By the definition of equilibrium strategy, since

$$\wp(A_k; A_1, \ldots, A_{k-1}, A_{k+1}, \ldots, A_n) = 0$$

for all k, we define strategies

$$B_k(x_j) := \sum_{\ell \neq k} A_\ell(x_j),$$

that all satisfy

$$\wp(B_k; B) \geq 0$$

for all strategies B as in Definition 1. We therefore have that these strategies B_k are equilibrium strategies for the two-player game. By the characterization of these strategies in the two-player game, the sum of equilibrium strategies is also an equilibrium strategy. Hence

$$\sum_{k=1}^{n} B_k = (n-1) \sum_{k=1}^{n} A_k$$

is an equilibrium strategy for the two-player game. Similarly, by the characterization of equilibrium strategies in the two-player game, if C_1 and C_2 are both equilibrium strategies, and $C_1(x_j) \geq C_2(x_j)$ for all j, and $|C_1 - C_2| > 0$, then $C_1 - C_2$ is an equilibrium strategy. Similarly, all positive constant multiples of an equilibrium strategy are an equilibrium strategy. We therefore have that

$$\sum_{k=1}^{n} B_k - (n-1)B_j = (n-1)A_j$$

is an equilibrium strategy, from which it follows that A_j is an equilibrium strategy for the two-player game, for each $j = 1, \ldots, n$. □
