# Supplementary material for: Biodiversity of marine microbes is safeguarded by phenotypic heterogeneity in ecological traits
Source: PLoS One. 2021 Aug 4;16(8):e0254799. doi: 10.1371/journal.pone.0254799 (PMC8336841; doi:10.1371/journal.pone.0254799)
Supplement: S2 Appendix — (ZIP) [file pone.0254799.s004.zip › S2_Appendix.pdf]

S2 Mathematical proofs for the continuous model. We first determine all equilibrium strategies for the continuous model when two strategies compete, and then we generalize to arbitrary numbers of competing strategies to complete the proof of Theorem 1 for the continuous model.

Proposition 5. *In the continuous model, a pair of strategies (f_1, f_2) is an equilibrium point if and only if both f_1 and f_2 are positive constants. Moreover, they satisfy*

$$\wp(f_i; g) \geq 0, \quad i = 1, 2$$

for all strategies g with equality if and only if $\mathrm{MCA}(g) = \frac{1}{2}$.

Proof. For any g competing with a positive constant function, denoted by $u(x) = U(1)$,

$$\wp(u; g) = \frac{1}{U(1) + G(1)} \int_0^1 U(1) \left(\int_0^x g(t)dt - \int_x^1 g(t)dt \right) dx$$

$$= \frac{U(1)}{U(1) + G(1)} \int_0^1 (G(x) - G(1) + G(x))\, dx = \frac{U(1)}{U(1) + G(1)} \left(\int_0^1 2G(x)dx - G(1) \right),$$

where $G(x) := \int_0^x g(t)dt$. We recall that the constraint requires

$$\frac{1}{G(1)} \int_0^1 xg(x)dx \leq \frac{1}{2}.$$

Integration by parts shows that this is equivalent to

$$\frac{1}{G(1)} \left(G(1) - \int_0^1 G(x)dx \right) \leq \frac{1}{2} \iff G(1) \leq 2 \int_0^1 G(x)dx.$$

Consequently, we obtain that

$$\wp(u; g) \geq 0,$$

with equality precisely when the MCA constraint is an equality, that is $\mathrm{MCA}(g) = \frac{1}{2}$.

We therefore obtain that for f_1 and f_2 as in the theorem,

$$\wp(f_1; f_2) = 0 = \wp(f_2; f_1).$$

Moreover, changing the strategy

$$\wp(g; f_2) = -\wp(f_2; g) \leq 0 = \wp(f_1; f_2)$$

for any strategy g, and similarly,

$$\wp(g; f_1) = -\wp(f_1; g) \leq 0 = \wp(f_2; f_1).$$

The pair (f_1, f_2) is therefore by definition an equilibrium point. To characterize all equilibrium strategies, we note that if (f, g) is an equilibrium point, then we must have

$$\wp(f; g) = \wp(g; f) = 0.$$

To see why this must be true, assume for the sake of contradiction that $\wp(f; g) > 0$. Then by the zero sum dynamic

$$\wp(g; f) < 0 = \wp(f; f).$$

This contradicts the definition of equilibrium strategy. Next, assume for the sake of contradiction that $\mathrm{MCA}(g) < \frac{1}{2}$. Then we have

$$\wp(f; g) = 0 < \wp(u; g),$$

contradicting the definition of equilibrium strategy. It follows that all equilibrium strategies must have $\mathrm{MCA} = \frac{1}{2}$. Moreover, since an equilibrium point (f_1, f_2) must satisfy

$$\wp(f_1; f_2) = \wp(f_2; f_1) = 0,$$

by definition of equilibrium strategy there can not exist a strategy g such that either

$$\wp(g; f_2) > 0 \text{ or } \wp(g; f_1) > 0.$$

To prove that all equilibrium strategies are positive constant functions, we will show that for any strategy f that is not a positive constant, we can construct a strategy g such that

$$\wp(g; f) > 0. \tag{10}$$

If $\mathrm{MCA}(f) < 1/2$ then $g(x) = G(1) > 0$ satisfies (10), so we may assume $\mathrm{MCA}(f) = 1/2$. Then, we have that

$$\wp(g; f) > 0 \iff \int_0^1 g(x)(2F(x) - F(1))dx > 0 \iff \int_0^1 g(x)\left(F(x) - \frac{F(1)}{2}\right)dx > 0$$

$$\iff \int_0^1 g(x)\left(\frac{F(x)}{F(1)} - \frac{1}{2}\right)dx > 0.$$

We will search for g that has $\mathrm{MCA}(g) = \frac{1}{2}$, consequently

$$\int_0^1 \frac{g(x)}{2}dx = \frac{G(1)}{2} = \int_0^1 xg(x)dx.$$

We therefore substitute obtaining

$$\wp(g; f) > 0 \iff \int_0^1 g(x)\left(\frac{F(x)}{F(1)} - x\right)dx > 0.$$

Since $\mathrm{MCA}(f) = 0.5$,

$$\int_0^1 xdx = \frac{1}{2} = \frac{1}{F(1)}\int_0^1 f(x)xdx = 1 - \int_0^1 \frac{F(x)}{F(1)}dx = 2\int_0^1 xdx - \int_0^1 \frac{F(x)}{F(1)}dx,$$

that is,

$$0 = \int_0^1 \left(\frac{F(x)}{F(1)} - x\right)dx.$$

Since f is not constant there exists $x \in [0, 1]$ such that $F(x) \neq xF(1)$. Thus, the integrand above must assume both positive and negative values, and in particular,

$$\exists \quad 0 \leq a < b \leq 1 \text{ such that } \frac{F(x)}{F(1)} - x > 0 \quad \forall x \in [a, b]. \tag{11}$$

If (a, b) is not fully contained in either $(0, 1/2)$ or $(1/2, 1)$, then by continuity, it is possible to split (a, b) into smaller intervals, one of which is fully contained in either

$(0, 1/2)$ or $(1/2, 1)$. We therefore assume without loss of generality that (a, b) is contained in either $(0, 1/2)$ or $(1/2, 1)$. First, assume $(a, b) \subset (1/2, 1)$. Define

$$g(x) = \begin{cases} 4M^2x, & x \in [0, 1/(2M)] \\ -4M^2x + 4M & x \in [1/(2M), 1/M] \\ 2N\frac{x-a}{b-a}, & x \in [a, (a+b)/2] \\ 2N\frac{b-x}{b-a}, & x \in [(a+b)/2, b] \\ 0, & \text{otherwise.} \end{cases} \tag{12}$$

The constants M, N will be determined to guarantee that $\mathrm{MCA}(g) = 0.5$, and $\wp(g; f) > 0$. We denote by I_1 and I_2 the integrals

$$I_1 = \int_a^b \left(\frac{F(x)}{F(1)} - x\right) g(x)dx, \qquad I_2 = \int_0^{1/M} \left(\frac{F(x)}{F(1)} - x\right) g(x)dx. \tag{13}$$

Since

$$\wp(g; f) > 0 \iff \int_0^1 g(x) \left(\frac{F(x)}{F(1)} - x\right) dx > 0,$$

and

$$\int_0^1 g(x) \left(\frac{F(x)}{F(1)} - x\right) dx = I_1 + I_2 \geq I_1 - |I_2|.$$

we wish to estimate I_1 from below and $|I_2|$ from above.

By continuity, there is a strictly positive constant R such that

$$\frac{F(x)}{F(1)} - x > R > 0 \quad \forall x \in [a, b].$$

Therefore, we have the estimate

$$I_1 \geq RN\frac{b-a}{2}.$$

By definition of F, we have the estimate

$$|F(x)| = \left|\int_0^x f(t)dt\right| \leq \frac{||f||_\infty}{M}, \quad \forall x \in [0, 1/M].$$

Above, $||f||_\infty$ is the standard supremum norm of f which is finite because f is continuous on the compact set $[0, 1]$.

By the triangle inequality

$$\left|\frac{F(x)}{F(1)} - x\right| \leq \frac{1}{M}\left(\frac{||f||_\infty}{F(1)} + 1\right), \qquad \forall x \in [0, 1/M].$$

Since

$$0 \leq g(x) \leq 2M, \qquad \forall x \in [0, 1/M].$$

Thus,

$$|I_2| \leq \int_0^{1/M} 2M\frac{1}{M}\left(\frac{||f||_\infty}{F(1)} + 1\right) dx = \frac{2}{M}\left(\frac{||f||_\infty}{F(1)} + 1\right).$$

To conclude,

$$I_1 - |I_2| \geq RN\frac{b-a}{2} - \frac{2}{M}\left(\frac{||f||_\infty}{F(1)} + 1\right). \tag{14}$$

We will choose M, N such that both $\wp(g; f) > 0$ and $\mathrm{MCA}(g) = 1/2$, so we begin by computing

$$G(1) = 1 + N\frac{b-a}{2} \implies \frac{1}{G(1)} = \frac{2}{2 + N(b-a)}.$$

Integrating $xg(x)$ gives

$$\mathrm{MCA}(g) = \frac{2}{2 + N(b-a)} \left(\frac{N}{b-a} \frac{(b-a)(b^2-a^2)}{4} + \frac{1}{2M} \right). \tag{15}$$

Set $\mathrm{MCA}(g) = 1/2$, that is,

$$\frac{1}{2} = \frac{2}{2 + N(b-a)} \left(N\frac{b^2-a^2}{4} + \frac{1}{2M} \right).$$

Therefore,

$$N = \frac{2}{(b-a)(a+b-1)} \left(1 - \frac{1}{M} \right). \tag{16}$$

Here it is important to observe that since $a < b$, and $[a, b] \subset [1/2, 1]$, $a + b - 1 > 0$, so we are not dividing by zero, and $N > 0$.

Inserting N from (16) into (14),

$$I_1 - |I_2| \geq R\frac{1}{a+b-1} \left(1 - \frac{1}{M} \right) - \frac{2}{M} \left(\frac{||f||_\infty}{F(1)} + 1 \right).$$

Consequently $I_1 > |I_2|$ is achieved by choosing M such that

$$2\left(\frac{||f||_\infty}{F(1)} + 1 \right) \frac{a+b-1}{R} + 1 < M. \tag{17}$$

This completes the proof in the case $(a, b) \subset (1/2, 1)$.

If $(a, b) \subset (0, 1/2)$, define

$$g(x) = \begin{cases} 2N\frac{x-a}{b-a}, & x \in [a, (a+b)/2] \\ 2N\frac{b-x}{b-a}, & x \in [(a+b)/2, b] \\ 4M^2(x - 1 + 1/M), & x \in [1 - 1/M, 1 - 1/(2M)] \\ 4M^2(1-x), & x \in [1 - 1/(2M), 1] \\ 0, & \text{otherwise,} \end{cases} \tag{18}$$

with constants M, N to be determined. First, we shall fix N so that the function g satisfies the MCA constraint. We compute

$$G(1) = 1 + N\frac{b-a}{2}, \quad \mathrm{MCA}(g) = \frac{2}{2 + N(b-a)} \left(N\frac{b^2-a^2}{4} + 1 - \frac{1}{2M} \right).$$

We set this equal to $\frac{1}{2}$, and obtain the equation

$$N\frac{b^2-a^2}{4} + 1 - \frac{1}{2M} = \frac{1}{4}\big(2 + N(b-a)\big).$$

Re-arranging,

$$N\frac{b-a}{4}(b+a) - N\frac{b-a}{4} = \frac{1}{2M} - \frac{1}{2},$$

hence we require that

$$N = \frac{2}{b-a}\frac{1 - 1/M}{1 - a - b}. \tag{19}$$

In this case, it is important to note that $(a, b) \subset (0, 1/2)$, so the denominator is positive. We shall choose

$$M > 1. \tag{20}$$

We denote by I_1 and I_2 the integrals

$$I_1 = \int_a^b \left(\frac{F(x)}{F(1)} - x \right) g(x)dx, \qquad I_2 = \int_{1-1/M}^1 \left(\frac{F(x)}{F(1)} - x \right) g(x)dx.$$

By continuity, there is a strictly positive constant R such that

$$\frac{F(x)}{F(1)} - x > R \quad \forall x \in [a, b].$$

Therefore, we have the estimate

$$I_1 \geq RN\frac{b-a}{2}.$$

Next, we wish to estimate $|I_2|$ from above. For this we note that

$$\left| \frac{F(x)}{F(1)} - x \right| = \left| \frac{F(x) - F(1) + F(1)(1-x)}{F(1)} \right|$$

$$\leq \frac{|F(x) - F(1)|}{F(1)} + |1-x| = \frac{1}{F(1)} \int_x^1 f(t)dt + |1-x| \leq |1-x| \left(\frac{||f||_\infty}{F(1)} + 1 \right).$$

Above, $||f||_\infty$ is the supremum norm of f, that is finite because f is continuous on the compact set $[0, 1]$. Consequently, since $0 \leq g(x) \leq 2M$ for all $x \in [1 - 1/M, 1]$, and $0 \leq |1 - x| \leq \frac{1}{M}$ on this interval, we estimate

$$|I_2| \leq \int_{1-1/M}^1 (2M)\frac{1}{M} \left(\frac{||f||_\infty}{F(1)} + 1 \right) dx \leq \frac{2}{M} \left(\frac{||f||_\infty}{F(1)} + 1 \right).$$

We thus obtain the estimate

$$I_1 + I_2 \geq I_1 - |I_2| \geq RN\frac{b-a}{2} - \frac{2}{M} \left(\frac{||f||_\infty}{F(1)} + 1 \right).$$

Eliminating N using (19), we obtain an estimate that can be controlled by the value of M, namely

$$I_1 + I_2 \geq R\frac{1 - 1/M}{1 - a - b} - \frac{2}{M} \left(\frac{||f||_\infty}{F(1)} + 1 \right).$$

We therefore have

$$R\frac{1 - 1/M}{1 - a - b} - \frac{2}{M} \left(\frac{||f||_\infty}{F(1)} + 1 \right) > 0$$

$$\Longleftrightarrow$$

$$M > 1 + 2\frac{1 - a - b}{R} \left(\frac{\|f\|_\infty}{F(1)} + 1 \right).$$

Notice that this condition on M guarantees that (20) is satisfied. Consequently,

$$I_1 + I_2 > 0 \implies \wp(g; f) > 0.$$

□

We can now complete the proof of Theorem 1 for the continuous model.

Proof of Theorem 1 for the continuous model

Proof. Assume that $\{f_k\}_{k=1}^n$ is a set of positive constant functions. We first prove that this set of strategies is an equilibrium point. Consider any strategy g as in Definition 2. Then if we change strategy f_1 to g, the resulting payoff $\wp(g; f_2, \ldots, f_n) =$

$$\frac{1}{G(1) + \sum_{k\geq 2} F_k(1)} \int_0^1 g(x) \left[\int_0^x \left(g(t) + \sum_{k\geq 2} f_k(t) \right) dt - \int_x^1 \left(g(t) + \sum_{k\geq 2} f_k(t) \right) dt \right] dx.$$

However, by the assumption on f_k in the theorem and the zero-sum dynamic, we have

$$\wp(f_k; g) \geq 0 \implies \wp(g; f_k) \leq 0 \implies \int_0^1 g(x) \left[\int_0^x f_k(t)dt - \int_x^1 f_k(t)dt \right] dx \leq 0,$$

$\forall k = 2, \ldots, n$. Therefore, since the internal competition within g does not affect the payoff, we have

$$\int_0^1 g(x) \left[\int_0^x \sum_{k=2}^n f_k(t)dt - \int_x^1 \sum_{k=2}^n f_k(t)dt \right] dx \leq 0 \implies \wp(g; f_2, \ldots, f_n) \leq 0. \quad (21)$$

By the assumptions of the theorem we have for all pairs f_j and f_k both

$$\wp(f_k; f_j) \geq 0, \quad \wp(f_j; f_k) \geq 0.$$

Due to the zero sum dynamic

$$\wp(f_k; f_j) = -\wp(f_j; f_k) \implies \wp(f_k; f_j) = 0 \quad \forall j, k \in \{1, \ldots, n\}.$$

Consequently, we have

$$\int_0^1 f_k(x) \left[\int_0^x f_\ell(t)dt - \int_x^1 f_\ell(t)dt \right] dx = 0, \quad \forall k, \ell \in \{1, \ldots, n\},$$

and therefore

$$\wp(f_k; f_1, \ldots, f_{k-1}, f_{k+1}, f_n) = 0 \quad \forall k = 1, \ldots, n.$$

By (21), we therefore have $\wp(g; f_2, \ldots, f_n) \leq \wp(f_1; f_2, \ldots, f_n) = 0$. The same argument applies to f_k for all k, namely, for any g as in, Definition 2,

$$\wp(g; f_1, \ldots, f_{k-1}, f_{k+1}, \ldots f_n) \leq 0 = \wp(f_k; f_1, \ldots, f_{k-1}, f_{k+1}, \ldots, f_n).$$

It follows that the set of strategies $\{f_k\}_{k=1}^n$ is an equilibrium point.

To prove the converse, we begin by assuming that $\{f_k\}_{k=1}^n$ is an equilibrium point. For the sake of contradiction, let us see what would happen if

$$\wp(f_1; f_2 \ldots, f_n) < 0 \implies \int_0^1 f_1(x) \left[\int_0^x \sum_{k=2}^n f_k(t)dt - \int_x^1 \sum_{k=2}^n f_k(t)dt \right] dx < 0.$$

Defining a new strategy g_1 so that

$$g_1(x) := \sum_{k=2}^n f_k(x) \implies \text{MCA}(g_1) \leq \frac{1}{2},$$

and

$$\int_0^1 g_1(x)\left[\int_0^x \sum_{k=2}^n f_k(t)dt - \int_x^1 \sum_{k=2}^n f_k(t)dt\right] dx = 0$$

$$\implies \wp(g_1; f_2, \ldots, f_n) = 0 > \wp(f_1; \ldots, f_n),$$

contradicting the definition of equilibrium strategy. We therefore have that $\wp(f_1; f_2, \ldots, f_n) \geq 0$, and the same argument shows that $\wp(f_k; f_1, \ldots, f_{k-1}, f_{k+1}, \ldots f_n) \geq 0$ for all $k = 1, \ldots, n$. By the zero-sum dynamic, the sum over all of these payoffs must vanish, hence each of these inequalities must in fact be an equality, so all these payoffs are zero. By the definition of equilibrium strategy, we must have that for any strategy g,

$$\wp(g; f_2, \ldots, f_n) \leq \wp(f_1; f_2 \ldots, f_n) = 0$$
$$\implies \int_0^1 g(x)\left[\int_0^x \sum_{k=2}^n f_k(t)dt - \int_x^1 \sum_{k=2}^n f_k(t)dt\right] dx \leq 0.$$

By the definition of equilibrium strategy, since $\wp(f_k; f_1, \ldots, f_{k-1}, f_{k+1}, \ldots, f_n) = 0$ for all k, we define strategies

$$g_k(x) := \sum_{\ell \neq k} f_\ell(x),$$

that all satisfy

$$\wp(g_k; g) \geq 0$$

for all strategies g as in Definition 2. We therefore have that these g_k are equilibrium strategies for the two-player game. Consequently, they are all positive constant functions. It follows that

$$\sum_{k=1}^n g_k = (n-1)\sum_{k=1}^n f_k$$

is also a positive constant function as is

$$\sum_{k=1}^n g_k - (n-1)g_j = (n-1)f_j,$$

for each $j = 1, \ldots, n$. It immediately follows that each f_j is a positive constant function for $j = 1, \ldots, n$. □
